# Lipophagy: Connecting Autophagy and Lipid Metabolism

**DOI:** 10.1155/2012/282041

**Published:** 2012-03-28

**Authors:** Rajat Singh, Ana Maria Cuervo

**Affiliations:** ^1^Department of Medicine, Albert Einstein College of Medicine, Bronx, NY 10461, USA; ^2^Department of Molecular Pharmacology, Albert Einstein College of Medicine, Bronx, NY 10461, USA; ^3^Diabetes Research Center, Albert Einstein College of Medicine, Bronx, NY 10461, USA; ^4^Department of Developmental and Molecular Biology, Albert Einstein College of Medicine, 1300 Morris Park Avenue, Bronx, NY 10461, USA; ^5^Institute for Aging Studies, Albert Einstein College of Medicine, Bronx, NY 10461, USA

## Abstract

Lipid droplets (LDs), initially considered “inert” lipid deposits, have gained during the last decade the classification of cytosolic organelles due to their defined composition and the multiplicity of specific cellular functions in which they are involved. The classification of LD as organelles brings along the need for their regulated turnover and recent findings support the direct contribution of autophagy to this turnover through a process now described as lipophagy. This paper focuses on the characteristics of this new type of selective autophagy and the cellular consequences of the mobilization of intracellular lipids through this process. Lipophagy impacts the cellular energetic balance directly, through lipid breakdown and, indirectly, by regulating food intake. Defective lipophagy has been already linked to important metabolic disorders such as fatty liver, obesity and atherosclerosis, and the age-dependent decrease in autophagy could underline the basis for the metabolic syndrome of aging.

## 1. Introduction

Autophagy, or the process of degradation of intracellular components in lysosomes, has been traditionally linked to cellular energy balance and to the cellular nutritional status [1, 2]. In fact, although during the recent revival of the autophagic process, most of the emphasis has been placed on its role in other cellular functions such as cellular quality control, remodeling, or cell defense, the first descriptions of the autophagic process in the early 1960s already stated that conditions such as starvation lead to its activation [3–5]. These early studies proposed that autophagic activation during starvation was necessary to maintain the cellular energetic balance. Later studies in yeast, in fact confirmed that activation of autophagy was essential to preserve cellular viability during nutritional starvation (nitrogen depletion in yeast), and that mutants defective in autophagy were lethal [6, 7]. In most of these studies emphasis was placed on the ability of autophagy to supply through degradation of protein products the amino acids required to maintain protein synthesis under the extreme nutritional conditions.

However, the contribution of autophagy to the cellular energetic balance may not be solely dependent on this capacity to provide free amino acids, which in fact, are a relatively inefficient source of energy when oxidized to urea and carbon dioxide. Recent studies support that autophagy can also provide energetically more efficient essential components, such as free fatty acids (FFAs) and sugars. In this paper, we focus on the contribution of autophagy to lipid catabolism and the consequences of this novel autophagic function in the cellular energetic balance as well as in specific lipid-mediated regulatory functions. Lastly, we also discuss the possible implications of alterations in the autophagic breakdown of lipids in human health and disease, with emphasis on common metabolic disorders.

## 2. Mobilization of Lipid Droplets by Autophagy

### 2.1. The Upgrading of Lipid Droplets to the Organelle Category

**Figure 1:**
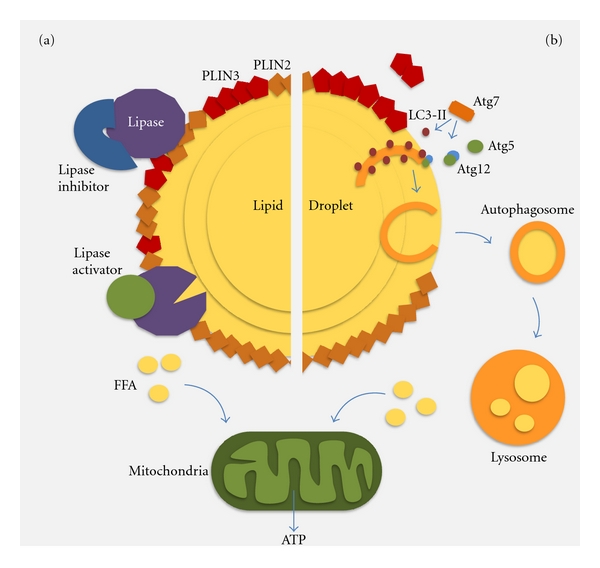
Lipolysis of lipid droplets. (a) Schematic representation of the main lipid and protein components of lipid droplets (LDs) and mechanisms of lipid mobilization (lipolysis) by cytosolic lipases. (b) Lipolysis by lipophagy. Schematic representation of the formation of autophagic vacuoles at the surface of an LD. PLIN: perilipin; Atg: autophagy-related protein; FFA: free fatty acids.

Cells store fat in the form of lipid droplets (LDs)—intracellular deposits of lipid esters surrounded by a monolayer of phospholipids and separated from the hydrophilic cytosolic environment by a coat of structural proteins, known generically as perilipins (Figure 1) [8, 9]. Despite their misleading appearance of inert stores, studies during recent years have revealed that LD are sites of high activity and that their functions are not limited to passive store of lipids. In fact, their dynamic nature, multifunctionality, and defined identity have now conferred upon them the category of intracellular organelles [8, 9]. Furthermore, as many other organelles, LDs have been shown to interact in a regulated manner with other intracellular compartments (likely, to provide them with specific lipids for their membranes) and to adapt to changes in the cellular environment [10, 11]. A growing theme in the field of LD research is now the identification of functions for the LD beyond those related to lipid metabolism or supply of membrane lipids. Pioneering among those has been the finding that the hydrophobic matrix of the LD can become a sequestering surface for misfolded proteins that if left free in the cytosol could organize into oligomeric and aggregated products highly toxic for cells [12, 13]. LD sequestration of proteins does not only apply to pathogenic proteins destined for degradation, but also may have a regulatory role in the availability of some fully functional proteins. For example, certain histones elude nuclear translocation through dynamic and reversible interactions with LD [14]. Interestingly, pathogens such as some types of viruses, have found in the LD ideal platforms for assembly [15, 16].

### 2.2. Mobilization of Intracellular Lipids through Lipolysis: Novel Role of Autophagy

Although lipid droplets are particularly prominent in the adipose tissue—where they organize as a single large droplet (up to 100 *μ*m diameter) that occupies almost the totality of the cytoplasm—all cells contain lipid droplets to variable extents that range from 0.1 to 10 *μ*m. In addition to size differences, the adipose tissue LD has a core predominantly formed by triglycerides (TGs) whereas in most cells cholesterol and TG share the nuclear core of the LD [8, 9].

LD originate from the ER and maintain a close connection with this organelle, which facilitates exchange of lipids and proteins between both compartments to accommodate to the metabolic requirements of the cell [10]. Mobilization of the lipids inside the LD occurs through lipolysis. The interaction of lipases present at the surface of the LD with the structural proteins that surround LD and with inhibitory proteins in the cytosol contributes to modulate the rate of lipolysis [17]. Cells activate lipolysis not only when they need energy but also in response to a large affluence of lipids to prevent stores from becoming compromisingly enlarged for the cell.

Although, traditionally, mobilization of LD by lipolysis has been solely attributed to the LD-associated lipases, recent studies have revealed a role for autophagy in LD breakdown (Figure 1). The presence of lipases in the lysosomal lumen, along with a large variety of hydrolases such as proteases, glycases and nucleases, has been acknowledged since the early days of the discovery of this organelle. However, lysosomal lipases, also known as “acid lipases” because of their optimal acidic pKa, were thought to serve mainly in the degradation of lipids contributed by the diet through endocytosis or those present in the membranes of the organelles digested during the autophagic process. The elevation of LD to the category of cytosolic organelles was in part a motivation to address their turnover by autophagy. This catabolic process is capable of sequestering whole cytosolic organelles inside double-membrane vesicles known as autophagosomes, which deliver this cargo to lysosomes upon heterotypic fusion with their membrane [18, 19]. Each of the events of the autophagic process is coordinated by a complex network of more than 32 genes and their protein products (autophagy-related genes (ATGs) and proteins (Atgs)). Atgs participate at the level of: (1) activation, (2) nucleation of the autophagosome membrane that forms *de novo* through conjugation of proteins and lipids from different cellular compartments, (3) elongation of the membrane and sealing to form the autophagosome, (4) trafficking toward the lysosomes, and (5) fusion of the two membranes [18].

The first hint that LD could become substrates of the autophagic process originated from studies in cultured hepatocytes knocked down for Atg5, one of the genes essential for the formation of autophagosomes [20]. Hepatocytes respond to an acute oleic challenge by increasing lipolysis, which would prevent massive enlargement of the LD compartment. Oleic challenge resulted in a marked increase in the number and size of LD in cells with compromised macroautophagy. The same was true *in vivo*, when knockout in liver of another essential autophagy gene (Atg7) led to an accelerated development of liver steatosis (fatty liver) in the autophagy compromised animals, when compared to control animals [20]. Detailed biochemical and functional analyses helped in establishing that the observed lipid accumulation did not result from increased formation of LD or reduced lipid secretion from hepatocytes, but that, instead, it could be explained almost exclusively on the basis of reduced lipolysis [20].

It is possible that, through mechanisms yet to be identified, changes in autophagic activity may modulate the LD-associated lipases and contribute to the observed changes in lipolysis. However, independent of this possibility, there is now evidence that the autophagic system contributes directly to the mobilization of lipids from LD to lysosomes, wherein luminal lipases mediate their lipolysis. In fact, neutralization of the lysosomal pH, that would have a marked effect on the lysosome-resident lipases but does not modify the activity of the cytosolic lipases, was enough to almost completely block the lipolysis activated in response to a lipid challenge [20].

### 2.3. Recognition of LD by the Autophagic System

Sequestration of cytosolic components inside the forming autophagosome was considered for a long time a nonselective in-bulk process by which cytosolic material was randomly delivered for lysosomal degradation. However, recent years have revealed the existence of a growing number of proteins dedicated to the tagging and recognition of cytosolic components for autophagic degradation [21]. In the case of intracellular protein aggregates, the presence of polyubiquitinated chains formed through specific types of linkage (the best characterized uses the lysine 63 in ubiquitin to link one ubiquitin moiety to another in the polyubiquitin chains) is the tag identified by the cargo-recognition machinery [22]. Similarly, ubiquitination of proteins on the surface of peroxisomes and of different pathogens contribute to their segregation towards the autophagic system [23, 24]. However, ubiquitin is not the only signal identified as marker for autophagic degradation. Selection of mitochondria for mitophagy (selective degradation of mitochondria by autophagy) has been shown to occur through different mechanisms, which likely coexist in most cells. In most cases, changes in structural components of the mitochondrial membrane are identified by partner cytosolic proteins (such as Parkin or Nix), that once bound at the surface of this organelle, tag it for degradation [25]. All cargo recognition molecules or autophagy receptors share their ability to bind to the tagging molecule in the organelle to be degraded as well as to specific components of the autophagic machinery (in almost all cases the light chain protein 3 or LC3) [21, 26]. This recruitment of autophagic components toward the cytosolic material to be degraded is proposed to initiate the *in situ* formation of the autophagosome around this material and to mediate selectivity.

Some levels of lipophagy may always occur even during the random sequestration of cytosolic material by “in-bulk” autophagy. In fact, analysis of the components inside autophagosomes in cells maintained in basal conditions revealed the presence of lipid material and LD structural proteins inside these vesicles, along with other cytosolic material [20]. However, as described in the previous section, when lipophagy is activated in response to a lipid challenge or prolonged starvation, there seems to be a switch toward the preferential sequestration of LD, supporting some level of selectivity in this process [20]. An intriguing observation in the studies of hepatic lipophagy was the fact that LD do not always seem to be sequestered as a “whole” by the autophagosomes, but, on the contrary, in many instances, only fractions of the LD underwent autophagy (Figure 1) [20]. Thus, membranous structures enriched in LC3, in support of their autophagic origin, appear to grow from the surface of the droplet towards the inner core. Often these membranes curve to finally seal, giving rise to double-membrane vesicles of a slightly smaller size than a conventional autophagosome (50–100 nm) and contain only components of the LD in their lumen. Interestingly, formation of the membranes seems to be polarized in only one site on the surface of the LD. Other components of the autophagic machinery, such as Atg5 and Atg7, also localize to these areas of the LD in further support that formation of the limiting membrane occurs at the surface of the LD (Figure 1) [20].

In the process of *de novo* formation of the autophagic membrane, Atg7 acts as the enzyme regulating conjugation of Atg12 to Atg5 (to serve as scaffold for assembling other components of the forming membrane), as well as the conjugation of LC3 to a lipid (phosphatidylethanolamine, PE) to generate LC3-II that is one of the best characterized structural components of the autophagosomes [18]. Interestingly, Atg7 is not required for the recruitment of LC3 to the LD, since this protein, although in its nonconjugated form, is still found associated to LD in cells defective in Atg7 [20]. PE, the only lipid known to conjugate to LC3, is among the phospholipids that contribute to form the delimiting phospholipid monolayer of LD [27]. The specific mechanism by which the limiting autophagosome membrane grows from the LD surface is still poorly characterized, but the presence of membrane-like structures in the LD core has been previously described [28]. In addition, although the core is predominantly composed of lipids, some proteins can also be detected in this region. Early studies have suggested that these proteins may form complexes with phospholipids to form structures compatible with the hydrophobic environment inside the LD [29]. In this respect, conjugation of LC3 to PE on the surface of the LD may provide the right conformation for the forming membrane to advance towards the inside of the LD.

The determinants for autophagic initiation on the surface of a LD remain unknown. Polyubiquitination has been detected in polarized areas of LD in part resulting from the accumulation of clusters of polyubiquitinated apolipoprotein B (ApoB) on their surface [13]. The fate of ApoB in this location seems to be to undergo lysosomal degradation. Whether or not the lysosomal degradation of ApoB occurs as a result of the activation of lipophagy and how the accumulation of this protein contributes to the initiation of the process requires future investigation. Recent studies have shown now the integration into the LD surface of the ancient ubiquitous protein 1 (AUP1), which bears a c-terminus able to bind enzymes involved in ubiquitination [30]. Whether or not the presence of AUP1 in LD is necessary or precedes the arrival of the autophagic machinery requires future investigation.

Of particular interest is the fact that LDs have been shown to dynamically interact with two of the organelles that have been proposed as sites of formation of the limiting membrane of the autophagosomes—the ER and the mitochondria (Figure 1) [11, 31]. Interactions with the ER may be related to LD biogenesis, as this is the compartment from where these organelles originate, but may also favor the distribution of lipids from the LD towards other organelles through the endosecretory pathway. In the case of mitochondria, the close interaction between LD and the outer-membrane of this organelle could facilitate delivery of the FFA released by lipolysis for mitochondrial *β*-oxidation. However, considering the described association of the autophagic initiation complex to punctual areas in the membrane of the ER and the mitochondria, and the formation of cup-like precursors of the limiting membrane of the autophagosomes from these regions [32, 33], it is tantalizing to at least propose that the previously described interactions of LD with these organelles, could also contribute to the initiation of their autophagic degradation.

## 3. Physiology of Lipophagy

### 3.1. Liver Lipophagy

**Figure 2:**
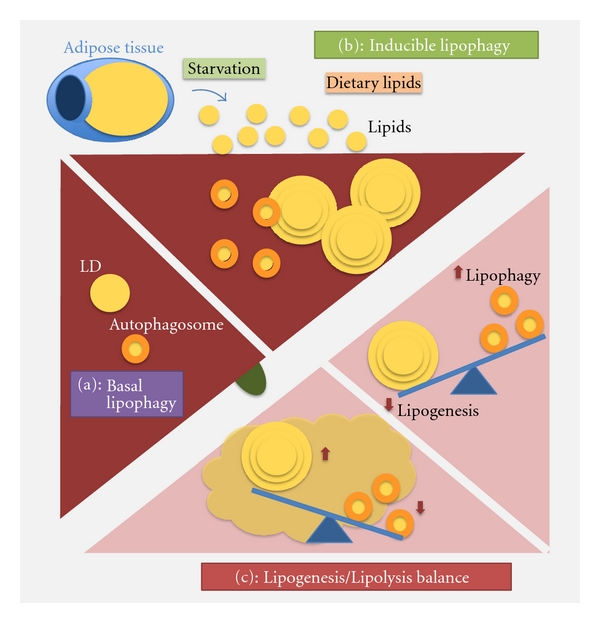
Autophagy and lipid metabolism in liver. (a) Basal lipophagy: some level of mobilization of LD by autophagy occurs continuously in all tissues including the liver. (b) Inducible lipophagy: stimuli such as prolonged starvation or maintained lipid challenges induce liver lipophagy to regulate LD growth. Failure to upregulate autophagy under these conditions could results in liver steatosis. (c) Lipogenesis/lipolysis balance: autophagy may also contribute to LD formation by mechanisms still unknown. A partial blockage of the autophagic process may modify the lipogenic-lipolytic balance in one direction or another depending on the cellular conditions.

As described in previous sections, mobilization of LD by autophagy was first observed both in cultured hepatocytes in response to fatty acid exposure, and in liver of mice maintained on a diet enriched in fat for prolonged periods of time (4 months) [20]. The liver responds to the massive influx of lipids from the blood by upregulating LD biogenesis, as a mechanism of defense against the toxicity of FA, which upon esterification get converted into TG and stored into LD [17]. However, in order to prevent uncontrolled expansion of LD, activation of lipolysis also occurs under these conditions and contributes to maintain LD size. Failure to regulate lipid accumulation in hepatocytes may be the basis of pathogenic conditions such as liver steatosis and steatohepatitis [8]. Autophagy has now been added to the mechanisms that control the growth of the hepatic LD under these conditions (Figure 2).

Besides lipid challenges, other stimuli such as starvation also engage the lipolytic contribution of the autophagic system. Classic measurement of protein catabolism in liver during starvation, revealed that most of the protein degradation in this organ occurs during the 4–6 h that follow starvation, and that protein breakdown, even of autophagic origin, decreases markedly once the 8–10 h of starvation are reached [4]. However, this decrease in autophagic degradation of proteins by autophagy is not equivalent to a decrease in overall autophagic activity. Formation and clearance of autophagosomes seems to be maintained throughout the starvation period, but there is a consistent change in the type of cytosolic components sequestered in these vesicles [20]. Whereas cytosolic proteins and some organelles are the main cargo of the autophagic process during the early hours of starvation, as lack of nutrients persists, there is a gradual change towards preferential sequestration of lipid droplets inside autophagosomes (Figure 2) [20]. This selective autophagy of lipid stores, known now as lipophagy, accounts for a high percentage of the lipolysis occurring during prolonged starvation in liver.

Interestingly, although lipophagy is markedly upregulated in response to lipid challenges and during prolonged starvation, it is possible that a certain percentage of degradation of lipid stores in lysosomes occurs continuously in many cell types. Thus, blockage of autophagy through knockdown of any of the essential Atg in hepatocytes in culture, leads to a significant increase in the number of lipid droplets in these cells even when maintained under normal nutritional conditions and in the absence of any additional challenge [20]. Similar basal lipophagy has also been observed in cell types not typically known to store fat such as fibroblasts, macrophages, T cells, dendritic cells, lymphoblasts, glia, striatal cell lines, and even primary neurons [20, 34–36], although the relative contribution of autophagy to basal lipolysis may vary depending on the cell type. Further studies are necessary to determine the reasons behind the coexistence of the two different mechanisms for lipolysis—the one mediated by the cytosolic lipases and the one occurring through the autophagic system. It is possible that activation of one or the other may mainly lead to quantitative differences (i.e., lipophagy may be able to provide large amounts of FFA in shorter time). However, because the lysosomal lipases have been poorly characterized, it is also possible that the quality and type of the resulting lipolytic products differs between cytosolic and lysosomal lipases. Lastly, in light of the growing evidence in support of the heterogeneity of the cellular LD, it is also plausible that the two lipolytic systems target different subpopulations of LD.

### 3.2. Specialized Lipophagy in the Hypothalamus

As detailed earlier, lipophagy appears to contribute significantly to the mobilization of cellular lipids for provision of energy [2]. However, the identification of lipophagy in cell types other than those involved in lipid storage, such as in immune or neuronal cells, suggests that autophagic turnover of lipids is perhaps a generic mechanism for the utilization of cellular fat stores in diverse cell types. In fact, recent reports have now included two additional cell types; hypothalamic neurons [35] and macrophage foam cells [37], to the increasing list of cells where lipophagy has been shown to be present and functionally important.

**Figure 3:**
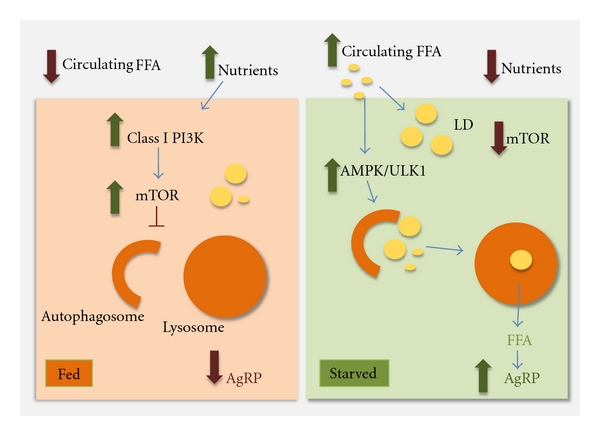
Conceptual model for hypothalamic lipophagy in control of food intake. In the fed state, active PI3K/mTOR signaling maintains autophagy at basal lower levels. Starvation increases circulating free fatty acids (FFAs), which activate hypothalamic autophagy by mechanisms that may in part require activation of AMPK/ULK1. These FFAs taken up by hypothalamic neurons are rapidly esterified into neutral lipids within lipid droplets. Activation of hypothalamic autophagy mobilizes neuronal lipids for the controlled availability of neuron-intrinsic FFAs that increase AgRP expression and food intake. AgRP: Agouti-related peptide, AMPK: AMP-activated protein kinase, FFA: free fatty acids, LD: lipid droplets, PI3K: phosphoinositide 3-kinase, mTOR: mammalian target of rapamycin, and ULK1: unc-51-like kinase 1.

The neurons within the mediobasal hypothalamus (MBH) form part of a focal neural network that integrates nutritional and hormonal information from two main cellular kinases, the mammalian target of rapamycin (mTOR) [38] and the phosphoinositol-3-kinase (PI3K) [39] to control food intake and energy balance [40]. Hypothalamic fatty acid metabolism, amongst other neuronal mechanisms, has been linked to the regulation of appetite [41, 42]. Although recent work suggests that neuronal FFA availability and oxidation provide the energetic requirements for activation and firing of orexigenic agouti-related peptide (AgRP) neurons [41], the lipolytic mechanisms that generate neuron-intrinsic FFA have remained poorly elucidated. Since autophagy is activated by starvation in most cells [43], it was plausible that autophagic mobilization of lipids in the hypothalamus could contribute to the generation of neuronal FFA during starvation that, in turn, trigger mechanisms driving food intake. In fact, a recent study in mice knockout for Atg7 in AgRP neurons shows that the hypothalamus, indeed, needs autophagy to upregulate expression of AgRP in response to nutritional depletion [35]. This indicates the distinctive characteristic of hypothalamic neurons in their ability to activate autophagy, quite unlike other regions of the brain in which autophagy does not seem to be under this type of nutritional regulation [1]. The activation of autophagy occurred in parallel to starvation-induced increases in hypothalamic FFA uptake (Figure 3), suggesting that, as in the case of the liver [20], acute FFA stimulus might be a mechanism for activation of hypothalamic autophagy during starvation. Indeed, the exposure of hypothalamic cells to FFA or FFA-rich serum from starved rodents increased autophagy (Figure 3) [35].

The activation of autophagy in hypothalamic neurons upon acute lipid stimulus associated with increases in the levels of phosphorylated AMPK and ULK1 [35], a kinase involved in the regulation of the autophagic process and recently described to be an AMPK substrate [44]. These findings indicate that hypothalamic AMPK and ULK1 may contribute to a FFA sensing mechanism that modulates autophagy in response to changes in nutrient signals. However, the mechanisms connecting FFA release and AgRP expression remain unknown for the most part. Although it is possible that FFA modulate some of the signaling cascades involved in AgRP regulation [45], a direct effect of the autophagic process on secretion of AgRP-containing vesicles cannot be ruled out.

The immediate fate of the FFA taken up by hypothalamic cells is esterification into neuronal LD, which underscored the requirement of a lipolytic mechanism to liberate neuronal FFA during starvation. Indeed, the activation of hypothalamic autophagy observed under these conditions, leads to increased mobilization of neuronal lipids to lysosomes [35]. The physiological consequence of these interactions is the generation of neuronal FFA, since inhibiting lysosomal hydrolysis or interfering with the autophagic process significantly decreases hypothalamic FFA levels. Lipophagy-generated hypothalamic FFA directly regulate the increase in orexigenic AgRP expression that occurs in AgRP hypothalamic neurons in response to starvation or to exposure to extracellular FFA [35]. In fact, selective blockage of autophagy in AgRP neurons has been shown to reduce fasting-induced increases in hypothalamic AgRP levels, food intake, and body weights. Interestingly, mice deficient in autophagy in AgRP neurons also displayed higher levels of the anorexigenic peptide *α*-melanocyte stimulating hormone (MSH), which is produced within the adjacent proopiomelanocortin (POMC) neuronal population, which could contribute to the reduced adiposity in these mice.

In this respect, it is interesting to note that the effect of autophagy in lipid mobilization and the downstream consequences of neuronal lipophagy may be very neuronal type-specific. For example, a recent study using acute intrahypothalamic injection of siRNA against Atg7 revealed increased adiposity and glucose intolerance in the injected mice [46], in contrast to the lean phenotype observed when only AgRP autophagy is compromised. It is possible that adiposity in this model occurred from concurrent reduction of atg7 in both AgRP and POMC neuronal populations, or from autophagic deficiency in additional cell types, for instance hypothalamic glial cells, also shown to regulate glucose homeostasis [47]. However, as the actual impact of the siRNA injection in the autophagic flux in this model is not known, is not possible to discard that differences in efficiency of the autophagic compromise between POMC and AgRP neurons are the real reason behind the different phenotype, or even that autophagy was not affected in the AgRP neurons and the phenotype only resulted from reduced POMC autophagy.

Although future investigation is required to clarify the cell type differences and to identify additional stimuli that may also modulate hypothalamic autophagy, the current findings highlight an exciting new role for lipophagy in control of food intake and whole body energy balance by modulating the “controlled production” of neuronal FFA that regulate AgRP levels [35] (Figure 3). In this way, the contribution of autophagy to the cellular and organismal energetic balance is no longer merely limited to its role in active breakdown of macromolecules or cellular stores to obtain energetic products, but has been raised to a more global regulatory function that includes the modulation of food intake. Forthcoming studies should help in addressing the possible implications of these findings for metabolic diseases such as obesity and the impact that these metabolic changes could have on hypothalamic autophagy.

### 3.3. Autophagy in the Adipose Tissue

The presence of lipophagy in diverse cell types raises the question whether lipophagy might also serve to mobilize lipids within the principal fat storing organ in the body, the adipose tissue. Surprisingly, recent reports by two independent groups demonstrate a completely new and unexpected function of autophagy in regulating adipose physiology [48, 49], which is quite distinct from the role of autophagy in mobilizing lipids observed in other cell types [20, 34–36]. In fact, adipose-selective knockout of essential autophagy genes in mouse significantly reduced adipocyte lipid droplet content and fat tissue mass [48, 49]. Likewise, blocking autophagy in cultured preadipocytes decreased cellular triglyceride content and levels of key adipogenic transcription factors CEBP-**α** (CCAAT/enhancer-binding protein *alpha*), CEBP-*β* (CCAAT/enhancer-binding protein *beta*) and PPAR-**γ** [48]. Conceivably, reduced expression of adipocyte-specific genes led to the formation of an adipose tissue that predominantly consisted of immature fat-deficient preadipocytes judging by their reduced levels of terminal differentiation markers (fatty acid synthase, fatty acid-binding protein-4 (FABP-4/aP-2), glucose transporter 4 (GLUT4), or stearoyl CoA desaturase 1) [48].

Intriguingly, selective inhibition of autophagy in white adipose tissue (WAT) *in vivo *not only impaired WAT differentiation but also introduced brown adipose tissue- (BAT-) like features in autophagy-deficient WAT [48]. The autophagy-deficient white adipocytes exhibited a cellular morphology that resembled closely that of brown adipocytes. For instance, atg7-deficient white adipocytes displayed increased number of smaller multiloculated lipid droplets and mitochondria, rounded nuclei, and larger cytoplasmic size [48, 49]. The molecular characteristics of the adipose tissue in the autophagy-deficient mice also mimicked those of BAT as reflected by increased levels of brown adipogenic factors, PPAR-**γ** transcriptional coactivator (PGC-1 **α**), and uncoupling protein-1 (UCP-1) [48]. Acquisition of BAT-like properties resulted in higher adipose tissue **β**-oxidation rates in knock-out animals than in controls [48, 49].

The physiological consequences of this shift in WAT phenotype were a decrease in body weight, reduced adiposity and resistance against high fat diet-induced alterations in glucose homeostasis [48, 49]. Although, the mechanism by which autophagy controls adipocyte differentiation or modulates the phenotypic switch from WAT to BAT-like is unclear, a likely possibility is that autophagy modulates levels of key regulatory proteins to control adipocyte cell fate, differentiation and fat storage. However, it is still plausible that specific autophagy-related components may be directly involved in the process of adipogenesis and that this function is not only limited to the adipose tissue. This concept is supported by studies performed at ages before adulthood in the same mouse model null for autophagy in liver used in the discovery of hepatic lipophagy [50]. In contrast to the massive accumulation of lipids observed in the adult animals, very young animals, at least when unchallenged, had consistently lower hepatocyte content of LD [50]. The previously described association of LC3-II to LD [20], also confirmed in this latter work, was proposed to be required for LD formation. Since expression of the enzyme used to flox out the autophagic gene does not start in this mouse model until 3-4 months after birth, it is possible that if autophagy is involved in both formation and mobilization of LD, the partial blockage of autophagy was initially sufficient to cope with the lipophagic requirements of the young animals and prevent the accumulation of LD. However, as the animals reached adulthood, the complete and persistent blockage of the autophagic system tilted the balance between lipogenesis and lipolysis toward the former one, leading to LD accumulation.

This dual involvement of autophagy in lipogenesis (LD formation) and lipolysis proposed in the liver now raises the question of whether a similar dual role could also occur in the adipose tissue. In fact, recent studies with a mouse model deficient in caveolin 1 have shown lipoatrophy of the adipose tissue mediated by massive upregulation of autophagy in this tissue [51]. Future studies with inducible autophagy knockouts in the adipose tissue of adult mice are needed to determine if autophagy does contribute to lipid mobilization also from fully formed adipose tissue.

## 4. Pathology of Lipophagy

### 4.1. Dual-Effect of Lipids on Autophagy

After the first observations demonstrating the existence of lipophagy and the upregulation of this process in response to a lipid challenge [20], numerous studies have confirmed the stimulatory effect of dietary lipids on the autophagic process. Upregulation of autophagy in response to increased FFA has been demonstrated in neurons, muscle, pancreas, mammary epithelial cells, liver-derived cells, and even in colon cancer cells [52–56]. Although the mechanisms that modulate the activation of the autophagic process under these conditions are still poorly elucidated, at least in the case of the pancreatic beta cell, autophagic activation has been proposed to occur through activation of the c-Jun N-terminal kinase 1 pathway in a manner independent of ER or oxidative stress [53].

**Figure 4:**
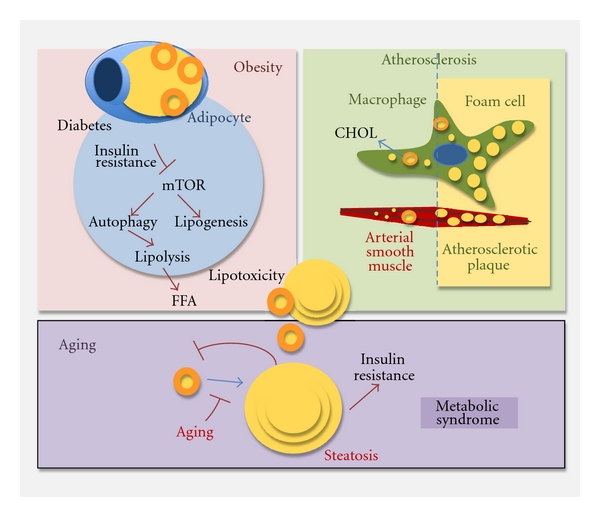
Lipophagy in pathology and aging. Alterations in the autophagic system and in its ability to mobilize intracellular lipids may underline the basis of important human disorders. The possible links of lipophagic malfunctioning with obesity, atherosclerosis, and the metabolic syndrome of aging are depicted.

In contrast to this stimulatory effect of a lipid challenge on the autophagic system, an equal number of studies have started to report inhibition of autophagy in response to exposure to high concentrations or particular type of lipids (Figure 4). For example unsaturated FFA such as oleic acid has a marked stimulatory effect on autophagy in many cells, at least up to some concentrations [20, 57, 58]. In contrast, saturated FFA such as palmitic acid—maybe due in part to its lower incorporation into LD—remains in the cytosol at higher concentrations and suppresses autophagy [58]. Likewise, in animals exposed to a high-fat diet for prolonged periods of time it is possible to detect an increase in autophagic activity during the first weeks of treatment, which is progressively followed by a gradual decrease in autophagy. This decrease further contributes to the expansion of the LD compartment, eventually leading to hepatotoxicity and steatosis [20, 57, 58]. Interestingly, the switch from activation to inhibition of autophagy in response to lipogenic stimuli can also be cell type dependent. Thus, same amounts of oxidized low-density lipoprotein that stimulate autophagic activity in schwannoma cells have been shown to be toxic for neuroblastoma cells [54].

Although many mechanisms could contribute to the inhibitory effect of FFA on autophagy, a systematic analysis of the different steps of the autophagic process has revealed a primary defect in the fusion between autophagosomes and lysosomes in cells exposed to high concentrations of FFA or in animals subjected to prolonged high-fat diet [57]. Interestingly, this failure to deliver autophagosome cargo directly to lysosomes is initially compensated for by increasing fusion of autophagic compartments with late endosomes (to generate what is known as an amphisome) [57]. However, as the high levels of intracellular lipids persist, defective intracellular turnover becomes evident, either because of further compromise of the pathway or maybe because of an additional failure in the endocytic system as autophagic cargo builds up in these compartments. Analysis of autophagic vacuoles from animals exposed to a high-fat diet revealed that changes in the lipid composition of the membrane of these vesicles are behind their compromised fusogenicity [57].

This-dual effect of dietary lipids on autophagy and lipophagy should be taken into consideration when contemplating manipulations of the autophagic system as a therapeutic strategy for metabolic disorders.

### 4.2. Liver Lipophagy and Hepatic Diseases

The fast development of steatosis and fatty liver observed in mice defective for autophagy in this organ [20] strongly supported the contribution of altered autophagy to the pathogenesis of this common disease. In fact, a compromise in hepatic autophagy has been proposed to underline also the basis for the accumulation of LD upon exposure to toxic concentrations of ethanol [59]. Furthermore, recent studies have demonstrated that pharmacological upregulation of autophagy reduces hepatotoxicity and steatosis in an alcohol-induced model of fatty liver [59]. However, future studies are needed before autophagy activation can be used as a generalized treatment against this disease, because, for example, upregulation of the autophagic process in hepatic stellate cells has been shown to favor their activation and consequently initiate liver fibrosis [60]. The liver responds to some stressors through global activation of autophagy, including degradation of lipids, proteins, and organelles. However, in other instances upregulation of autophagy as a protective mechanism against liver injury can be specific for lipophagy. For example, the autophagy upregulated as a first line of defense against alcohol-induced toxicity in liver, selectively targets mitochondria, and lipid droplets, while excluding soluble cytosolic proteins and other organelles [59]. Future efforts should focus in understanding how selective forms of autophagy can be individually modulated for therapeutic purposes.

The recently discovered capability of the autophagic system to mobilize hepatic lipids is also utilized by viruses to favor their replication. Earlier studies have shown that although autophagy is usually an efficient mechanism in the defense against most viral infections, upregulation of autophagy could also favor replication of some viruses such as the dengue virus. Recent studies have demonstrated that dengue virus-dependent induction of autophagy mediates LD breakdown and release of FFA necessary to maintain the high levels of intracellular ATP required for dengue viral replication [61]. Future studies are needed to determine which subset of hepatotropic virus makes use of lipophagy for their own replication and whether blockage of the autophagic system can be performed in this organ in a selective way to preferentially affect virogenesis but not normal liver metabolism.

### 4.3. Metabolic Disorders

The finding that autophagy is required for adipogenesis has elicited a considerable interest in the interplay between autophagy and metabolic disorders such as obesity.

Studies performed in human subjects with different types and degrees of obesity have revealed a direct correlation between autophagic activity and the sizes of various fat depots. Interestingly, autophagy was found to be inappropriately active in omental fat tissues extracted from obese individuals, and, in fact, autophagic activity was remarkably raised in insulin-resistant obese subjects [62]. This indicates that although functional autophagy may be a requirement for adipose differentiation during development, autophagy might also be involved in the maintenance of adipose tissue size and lipid storage in adults. The fact that autophagic upregulation occurs before obesity-associated morbidity becomes manifested, still leaves open the possibility that this system could be activated as a defensive mechanism against the increase in intracellular lipids. However, the final outcome and autophagy effect may be very different depending on the metabolic status. For example, in adipocytes from type 2 diabetes patients characterized by unresponsiveness to insulin, maintained attenuation of mTOR has been recently described and proposed as the main mechanism responsible for the upregulation of autophagy in these cells. The concomitant increase in LD formation under these conditions along with their enhanced autophagy favors cellular toxicity due to the excessive release of FFA from LD. It is anticipated that blockage of autophagy, or at least downregulation to a normal level, may be better under these conditions [63].

Lipophagy has been recently proposed as a possible defensive mechanism against atherosclerosis, or the thickening of the artery walls due to abnormal accumulation of lipid deposits in macrophage foam cells [37]. The recent finding that autophagy contributes to lipolytic mobilization of LD in macrophages also, provides now a new possible mechanism for the pathogenesis of atherosclerosis. This study has revealed that macrophage lipophagy is upregulated both *in vitro* and *in vivo* in response to lipid loading and that failure to upregulate the autophagic system, in mice defective for this pathway, results in inefficient clearance of cholesterol in macrophages [37]. In light of the inhibitory effect that high concentrations of intracellular lipids can have on autophagy [57], it is reasonable to propose that chronic exposure to high levels of circulating lipids may compromise the autophagic system of the artery wall macrophages and lead to their transformation into “foam cells” as lipids accumulate in their cytoplasm. This massive accumulation of lipids is the seeding for the subsequent formation of the atherosclerotic plaque. Current therapeutic strategies in this disease are aimed at promoting cholesterol efflux from these macrophages to reduce the size of the lipid-enriched plaque that they form beneath the endothelial cells. Consequently, manipulations aimed at enhancing macrophage autophagy and thus favoring cholesterol efflux from these cells, may have therapeutic potential in the atherosclerotic artery walls. Interestingly, the contribution of changes in autophagy to atherosclerotic plaque development may go beyond macrophages and involve also the smooth muscle cells of the arterial wall. Recent studies have shown compromised autophagy in these cells as a consequence of the inflammatory response associated to the plaques [64].

### 4.4. Aging

Autophagic activity decreases with age in most tissues and organisms [65]. The fact that a decline in autophagic activity, and in particular in lipophagy, would contribute to intracellular accumulation of LD and that, as described in previous sections, these abnormally expanded lipid stores would further reduce autophagic activity, makes this an attractive feedback loop for the perpetuation of the metabolic syndrome of aging (characterized by hypercholesterolemia, accumulation of lipid deposits in organs, and insulin resistance) (Figure 4). Interestingly, and in some way contra intuitively, treatment with antilipolytic agents has been shown to improve the age-related hypercholesterolemic phenotype and overall health-span in old mouse models [66]. However, recent studies support that most of the beneficial effect observed with these agents is dependent on their ability to induce autophagy, likely as a response to the increase in intracellular lipid stores [67].

Genetic connections among autophagy, lipid metabolism, and longevity have also been recently highlighted in studies in C. elegans [68]. Functional autophagy is necessary in this model to maintain the activation of a cellular lipase (LIPL-4) and conversely, this lipase is required for induction of autophagy. Interestingly, both the activity of this lipase and autophagy are required to attain the extension in life span observed upon germline removal in worms. Although the specific lipid targets of this lipase and the way in which it participates in autophagy remain unknown, it is tempting to propose that part of the effect in life-span could be due to better intracellular lipid handling by lipophagy.

## 5. Concluding Remarks

The recent discovery of lipophagy has contributed to link two major intracellular catabolic pathways autophagy and lipolysis. This new function of autophagy in lipid metabolism expands the physiological relevance of the autophagic process by making its contribution to the energetic balance more relevant (when considering the higher energetic value of lipids versus proteins), but also including now under the list of autophagic functions the control of many of the regulatory activities that lipids exert inside cells.

There are however a large number of standing questions that deserve immediate attention. Does lipophagy regulation occur through similar signaling pathways to those described for other types of autophagy? How are LDs selectively targeted by the autophagic machinery? Is there preferential degradation of a subset of LD by lipophagy? Are there differences between the types of lipid byproducts generated by lipophagy when compared to cytosolic lipolysis? What determines the threshold for the switch from a stimulatory to an inhibitory effect of FFA on lipophagy? And also, in a more general context, is upregulation of autophagy protective against all types of lipotoxicity *in vivo*? What are the possible effects of lipophagy on the combinations of metabolic defects that often coexist in our population such as obesity, diabetes, and hyperlipidemia? Does defective hypothalamic lipophagy with age contribute to the reduced food intake observed in advanced aging [69]?

Although these are still early days for lipophagy, there is now ample evidence that organ-specific targeting of this process may have implications for development of novel therapeutic interventions against common human metabolic disorders such as obesity and insulin resistance.
